# Experimental validation of a multinomial processing tree model for analyzing eyewitness identification decisions

**DOI:** 10.1038/s41598-022-19513-w

**Published:** 2022-09-16

**Authors:** Kristina Winter, Nicola M. Menne, Raoul Bell, Axel Buchner

**Affiliations:** Department of Experimental Psychology, Heinrich Heine University Düsseldorf, 40204 Düsseldorf, Germany

**Keywords:** Psychology, Human behaviour

## Abstract

To improve police protocols for lineup procedures, it is helpful to understand the processes underlying eyewitness identification performance. The *two-high threshold (2-HT) eyewitness identification model* is a multinomial processing tree model that measures four latent cognitive processes on which eyewitness identification decisions are based: two detection-based processes (the detection of culprit presence and absence) and two non-detection-based processes (biased and guessing-based selection). The model takes into account the full 2 × 3 data structure of lineup procedures, that is, suspect identifications, filler identifications and rejections in both culprit-present and culprit-absent lineups. Here the model is introduced and the results of four large validation experiments are reported, one for each of the processes specified by the model. The validation experiments served to test whether the model’s parameters sensitively reflect manipulations of the processes they were designed to measure. The results show that manipulations of exposure duration of the culprit’s face at encoding, lineup fairness, pre-lineup instructions and ease of rejection of culprit-absent lineups were sensitively reflected in the parameters representing culprit-presence detection, biased suspect selection, guessing-based selection and culprit-absence detection, respectively. The results of the experiments thus validate the interpretations of the parameters of the 2-HT eyewitness identification model.

## Introduction

Eyewitness identifications often are essential for convicting culprits. Given that eyewitness identification decisions are made after the crime has occurred, it is necessary to assess the witness’s memory to test the hypothesis that a suspect is the culprit. The memory test is usually done in the form of a lineup. Lineup procedures have to follow strict protocols^1^. The suspect’s face is to be presented among the faces of known innocent fillers. The witness’s task is to identify the culprit or reject the lineup. However, instead of correctly identifying the culprit or rejecting the lineup with an innocent suspect, the witness may falsely reject a lineup even though the culprit is present or select a known innocent filler. In the worst case, the witness identifies an innocent suspect^2^.

To improve police protocols for lineup procedures, it is helpful to understand the cognitive processes upon which these eyewitness identification decisions are based. Here we introduce the *two-high threshold (2-HT) eyewitness identification model* that measures four different latent processes underlying eyewitness identification performance: detection of the presence or absence of the culprit, biased suspect selection and guessing-based selection. To validate this model, we tested in four large experiments whether the four model parameters respond sensitively to manipulations that can be expected to influence the postulated latent processes represented by the parameters.

Many studies have examined how police lineup procedures affect the quality of eyewitness identification decisions^1,3^. At first glance, it may seem desirable that lineup procedures lead to a high rate of correct culprit identifications. However, this is so only if all culprit identifications are based on the detection of the culprit. Unfortunately, however, correct culprit identifications may also be caused by the biased selection of the suspect due to an unfair lineup procedure in which the suspect stands out from the fillers or by a tendency to select one of the lineup members based on guessing. Both of these processes do not only lead to correct culprit identifications but also to false innocent-suspect identifications. To understand eyewitness identification performance, it is thus important to distinguish between different latent processes that may contribute to eyewitness identification decisions.

To measure eyewitness identification performance, researchers have often relied on the *diagnosticity ratio*^3–9^ which is defined as the ratio between the proportion of correct culprit identifications and the proportion of false innocent-suspect identifications^10^. A higher diagnosticity ratio was taken to indicate superior eyewitness identification performance. However, the diagnosticity ratio attains higher values not only when a procedure is associated with better discrimination between culprits and innocent suspects but also when witnesses exhibit a conservative response bias^11–13^. Therefore, it has been suggested to apply *Receiver Operating Characteristic* (ROC) curves to the analysis of eyewitness identification decisions^11,13–18^. Derived from *signal detection theory (SDT)*^19^, ROC curves are constructed by plotting the hit rate (the proportion of correct detections of a signal) against the false alarm rate (the proportion of false positive responses to noise) at different levels of response bias. In lineup research, the hit rate corresponds to the rate of correct culprit identifications. The false alarm rate corresponds to the rate of false innocent-suspect identifications. The levels of response bias are usually derived from confidence judgements that witnesses are asked to assign to their identification decisions. To measure the degree to which the procedure allows witnesses to discriminate between culprits and innocent suspects, the *partial area under the curve* (*pAUC*) is calculated. The lineup procedure with the greater *pAUC* is to be preferred^11,20^.

The ROC analysis was derived from SDT^19^ that was originally proposed for detection problems with a 2 × 2 data structure (Table 1a): participants respond “yes” or “no” to a stimulus in which the signal is present or absent, respectively. There are four data categories in such tasks: hits, false alarms, misses and correct rejections. The analysis of performance is based on hits and false alarms because the remaining data categories are redundant (miss rate = 1 − hit rate; correct rejection rate = 1 − false alarm rate). By contrast, the detection problem in lineups has a 2 × 3 data structure (Table 1b) with other, non-redundant data categories. In culprit-present lineups, one may correctly identify the culprit (correct culprit identification), falsely reject the lineup (false lineup rejection) and falsely identify a filler (false filler identification). In culprit-absent lineups, one may falsely identify the innocent suspect (false innocent-suspect identification), correctly reject the lineup (correct lineup rejection) and falsely identify a filler (false filler identification).

**Table 1:** The data structures of the standard signal-detection task and the typical lineup identification task.

**The 2 × 2 data structure of the standard signal-detection task**			
	Yes		No
Signal present	Hit		Miss
Signal absent	False alarm		Correct rejection

**The 2 × 3 data structure of the typical lineup identification task**			
	Suspect identification	Filler identification	Lineup rejection
Culprit present	Correct culprit identification	False filler identification	False lineup rejection
Culprit absent	False innocent-suspect identification	False filler identification	Correct lineup rejection

It has been argued that, for the purpose of deciding which of two lineup procedures is superior, it is sufficient to focus only on correct culprit identifications and false innocent-suspect identifications because these data categories have the most far-reaching consequences in practice^11^. However, if the goal is to distinguish between the latent cognitive processes underlying eyewitness identification decisions, filler identifications can provide useful information. For instance, rejecting a culprit-absent lineup represents a correct decision whereas falsely identifying a filler represents an error. Hence, the underlying cognitive processes differ^8,9^. The 2-HT eyewitness identification model introduced here is thus based on the full range of data categories available from lineup procedures: correct culprit identifications, false innocent-suspect identifications, false filler identifications in culprit-present and culprit-absent lineups as well as correct and false lineup rejections. The model belongs to the class of multinomial processing tree (MPT) models that has proven useful to analyze, and to test hypotheses about, the latent processes underlying observable behavior in various fields of psychology, including memory (e.g.^21–24^) and decision making (e.g.^25–27^). There are several excellent introductions to MPT modeling^28,29^. Parameter estimation and statistical tests on the model parameters can be performed with freely available computer programs^30–32^.

**Figure 1:**
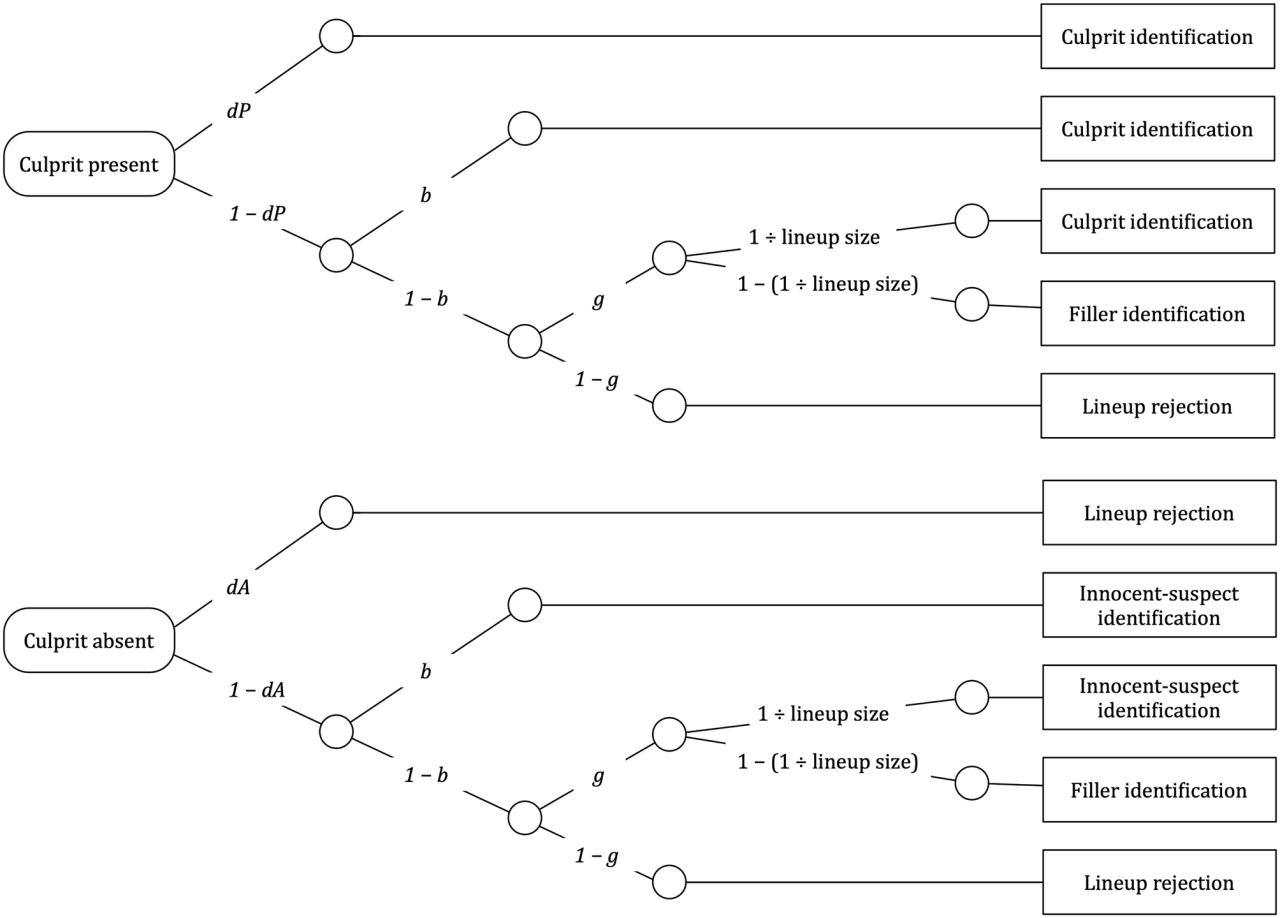
Graphical illustration of the two-high threshold (2-HT) eyewitness identification model. The rounded rectangles on the left represent culprit-present and culprit-absent lineups that have to be processed in order to arrive at an observable response. The rectangles on the right represent the observable response categories. The letters attached to the branches represent the probabilities of the latent cognitive processes postulated by the model. Parameter *dP* represents the probability of detecting the presence of the culprit. Parameter *b* represents the probability of biased selection of a suspect who stands out from the other lineup members. Parameter *g* represents the probability of guessing-based selection among the lineup members, as a consequence of which the suspect might be chosen based on chance with a probability of 1 ÷ lineup size (approximately 0.16667 in the present case of six lineup members). Parameter *dA* represents the probability of detecting the absence of the culprit*.* Identifiability was ensured using multiTree’s^30^ repeated analysis module in which the expectation–maximization algorithm is repeatedly applied to identical category frequencies (1000 times in the present case for every dataset presented here). Identical parameter estimates were obtained in all cases, confirming that the model is identifiable. There are as many free parameters as there are independent data categories to fit so that the unrestricted model has zero degrees of freedom.

A graphical illustration of the 2-HT eyewitness identification model is displayed in Fig. 1. The upper tree represents the processes leading to identifications and rejections in culprit-present lineups. The witness may detect the presence of the culprit with probability *dP* (for *detection of the presence of the culprit*). If culprit detection fails (with probability 1 − *dP*), the witness may identify the culprit based on two types of non-detection-based processes. If the lineup is unfair, the culprit stands out from the fillers so that it can be inferred who the suspect is without relying on memory. In such cases, biased selection of the suspect occurs with probability *b* (for *biased suspect selection*). With probability 1 − *b*, no biased suspect selection occurs (e.g., if the lineup is fair or the witness does not attend to the features creating the unfairness). In this case, it is still possible to select one of the lineup members as the culprit based on guessing. Guessing-based selection differs from biased suspect selection in that, when the witness selects one of the lineup members based on guessing, the culprit is selected among the fillers with a probability that is equal to 1 ÷ lineup size. With probability 1 − (1 ÷ lineup size), one of the fillers is selected. To illustrate, in a six-person lineup, the probability that selecting one of the lineup members based on guessing results in the identification of the suspect among the fillers is 1/6 while the probability of selecting one of the fillers is 5/6. The probability with which guessing leads to a suspect identification is a function of the size of the lineup and does not depend on the witnesses’ internal cognitive representation which is why this element of the model is a constant and not a parameter that has to be estimated from data. If none of the lineup members is selected based on guessing (with probability 1 − *g*), the lineup is falsely rejected.

For culprit-absent lineups in which the suspect is innocent (lower tree of Fig. 1), parallel cognitive processes are postulated to occur: the absence of the culprit and the fact that no one else in the lineup can possibly be the culprit is detected with probability *dA* (for *detection of the absence of the culprit*), leading to the correct rejection of the lineup. With probability 1 − *dA*, the absence of the culprit is not detected, in which case the same non-detection-based processes are postulated as in culprit-present lineups. An obvious characteristic of guessing-based selection (parameter *g*) is that it must not differ between culprit-absent and culprit-present lineups. The same applies to biased suspect selection (parameter *b*): the police do not know whether their suspect is the culprit or innocent. Therefore, if a lineup is unfair, it is equivalently unfair for culprits and innocent suspects. For reasons of ecological validity, it is thus optimal to have a designated innocent suspect who deviates from the fillers in a way that is similar to how the culprit deviates from the fillers when studying the effects of lineup unfairness.

The 2-HT eyewitness identification model is a two-high threshold model such as, for example, the widely used two-high threshold model of source memory^21,33^. The model includes parameter *dP* for the detection of the presence of the culprit and parameter *dA* for the detection of the absence of the culprit. While in other two-high threshold models the assumption that both detection parameters are equal is a requirement for identifiability (e.g.^21,34^), they can be estimated separately in the 2-HT eyewitness identification model (cf.^35^). Given that the model does not have to be restricted by setting the *dA* parameter equal to another parameter, it is possible to determine at an empirical level whether eyewitnesses are able to detect the absence of the culprit. To anticipate, the results of the present Experiment 4 and reanalyses of published studies^36^ suggest that people detect the absence of the culprit in some situations but fail to detect the absence of the culprit in others.

In a new MPT model it is necessary to validate the interpretation of the model parameters^28^. A successful validation requires that the model fits the empirical data and that the model parameters respond sensitively to manipulations of the processes they were designed to measure. For this purpose, validation experiments are needed in which well-established or outright trivial manipulations of the latent cognitive processes are used to test whether the changes in the latent cognitive processes are reflected in the model’s parameters. Here, we report four validation experiments, one for each of the four parameters of the model. To influence the detection of the presence of the culprit, reflected in parameter *dP*, we manipulated the exposure duration of the culprits’ faces at encoding. To influence biased suspect selection, reflected in parameter *b*, we manipulated the unfairness of the lineup. To influence guessing-based selection, reflected in parameter *g*, we manipulated whether the pre-lineup instructions suggested either a high or a low probability of the culprit being in the lineup. To influence the detection of the absence of the culprit, reflected in parameter *dA*, we compared the standard lineup procedure with a condition in which all lineup members in culprit-absent lineups could easily be ruled out as the culprit. Given the lively discussion about whether lineups should be simultaneous or sequential^11,13,37–39^, one group of participants saw simultaneous lineups while another group of participants saw sequential lineups in each of the four experiments.

## Experiment 1: Detection of the presence of the culprit (parameter *dP*)

Experiment 1 served to test the validity of parameter *dP* which is assumed to represent the probability of detecting the presence of the culprit. A culprit’s presence in a lineup is easier to detect when the culprit’s face was exposed for a long than for a short duration during a crime^40–42^. We thus presented long or short crime videos. If the 2-HT eyewitness identification model is valid, parameter *dP* should be higher in the long-exposure condition than in the short-exposure condition.

### Method

#### Sample

Participants were recruited using the *Gapfish* research panel (https://gapfish.com). Participants had to be of legal age (≥ 18 years) and had to have good eyesight and German language skills. A power analysis with *multiTree*^30^ showed that, given α = β = 0.05, at least *N* = 628 participants were needed to detect a difference in the *dP* parameter between the long- and short-exposure condition of Δ*dP* = 0.12 (corresponding to an effect size of *w* = 0.07), estimated from a pilot laboratory study. A somewhat larger sample was recruited to compensate for the loss of data that can be expected in online studies. In total, 932 participants gave informed consent, but 135 of them did not complete the study. A total of 33 data sets had to be excluded because of multiple participations (i.e., participants saw the crime video more than once). Another 22 data sets had to be excluded because participants had failed the attention check (see below) or because they had reported technical problems. The final sample included 742 participants (327 women and 415 men) with a mean age of 44 years (*SD* = 15).

#### Ethical approval

The following applies to all experiments reported here. Prior to participation, informed consent was obtained from all participants. Participants were warned that they would see a video containing verbal and physical abuse. They were asked not to continue if they felt uncomfortable when anticipating to view such a video. After the experiment, participants were informed about the purpose of the study and assured that the crime was staged. Ethical approval had been received from the ethics committee of the Faculty of Mathematics and Natural Sciences of Heinrich Heine University Düsseldorf. The study was run in accordance with the declaration of Helsinki. Informed consent for the publication was obtained from the actors of the staged-crime videos to show the stimulus material.

#### Materials and procedure

The experiment was conducted online using *SoSci Survey*^43^. Participants were only allowed to participate with computers or laptops, not with smartphones or tablets. Participants were asked to complete the study alone in a quiet environment. Prior to the experiment proper, participants were asked to bring their browsers into full-screen mode.

##### Staged-crime videos

**Figure 2:**
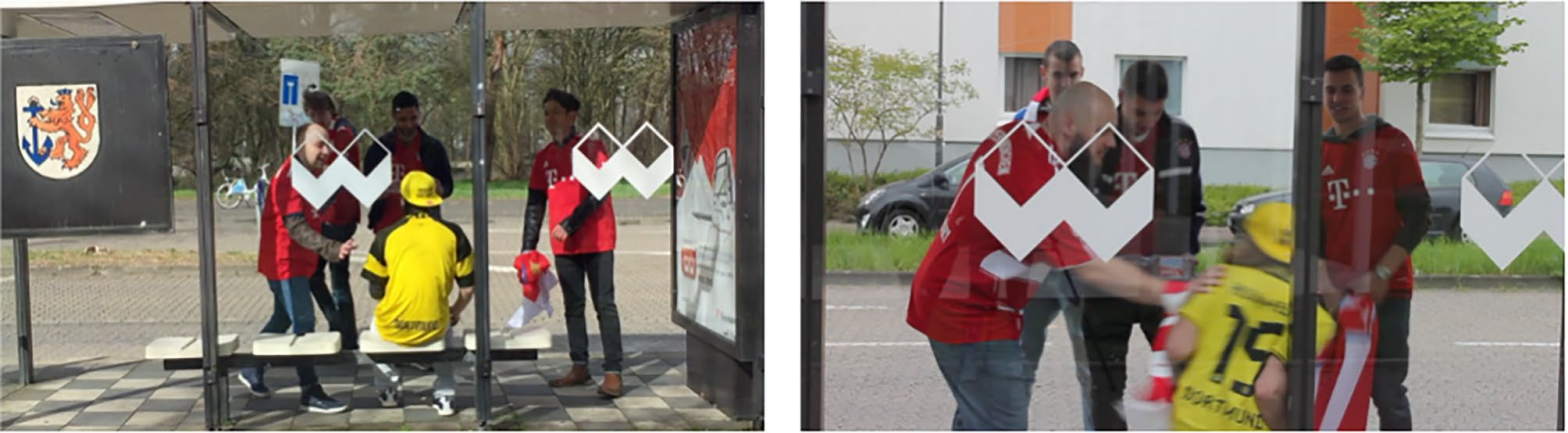
Parallel scenes from the two versions of the video. The videos depicted a staged crime. The culprits and the victim were volunteer actors.

In lineup research, the standard procedure involves exposing each participant to a single culprit at encoding and to a single (culprit-present or culprit-absent) lineup at test (see e.g.^13,44–48^). This procedure generates only a single data point per participant. In order to increase the efficiency of data collection, researchers have introduced between two and at least 14 to-be-identified persons in their lineup studies^41,49–58^. We followed this lead and presented our participants with a crime video containing four culprits. Specifically, the crime video showed four alleged hooligans of the German soccer club FC Bayern München attacking an alleged fan of a rivaling soccer club, Borussia Dortmund, at a bus station (Fig. 2). The culprits and the victim wore fan clothing (shirts, scarfs and caps) of their respective soccer clubs. The culprits insulted the victim, poked fun at him and tossed his belongings around. At the end of the video, the victim got knocked to the ground. The culprits continued to physically abuse the victim before they apparently noticed another person approaching (not visible in the video) and ran away shouting loudly.

Two parallel videos were created, henceforth called Video 1 and Video 2. The videos had the same content (i.e., the same verbal abuse and the same acts of violence in the same sequence and with the same timing), the important exception being that the victims and the culprits were different persons. However, care was taken that the victim in Video 1 was as similar as possible to the victim in Video 2 and that each of the four culprits in Video 1 was as similar as possible to one of the four culprits in Video 2 (i.e., Hooligan A in Video 1 matched Hooligan A in Video 2, Hooligan B in Video 1 matched Hooligan B in Video 2 and so on). It was randomly determined which of the two parallel versions of the video was shown. The videos were shown in a resolution of 885 × 500 pixels.

For each of the two parallel videos, we created a long version that lasted about 130 s and a short version that lasted only 13 s (the rest of the video was cut off). In both versions of the videos, the culprits’ faces were clearly visible from a frontal view. Participants were randomly assigned to one of the two exposure-duration conditions. About half of the participants saw the long video while the other half of the participants saw the short video.

Participants could start the video by clicking on a “Start” button. The control elements of the video player were disabled so that participants could not fast-forward, replay or stop the video. After the video had been presented, participants received an attention-check question about the persons they saw in the video. They passed the attention check when they were able to remember that “soccer fans” had been shown in the video by choosing this option among ten alternatives. The option “athletes” (intended to be one of the nine distractors) was frequently chosen instead of the nominally correct option which suggests that this option was unintentionally ambiguous. Therefore, selecting this option was counted as correct in Experiment 1. This option was replaced by the less ambiguous option “waiters” in all subsequent experiments.

##### Lineup procedures

Participants were informed that they had to identify the FC Bayern München hooligans from the video they had just seen. Participants received two-sided lineup instructions that emphasized both the need to identify the culprit if the culprit was present and the need to reject the lineup if the culprit was absent. Participants were not informed about how many lineups were about to follow.

Participants saw four separate lineups, two culprit-present lineups and two culprit-absent lineups, in random order. Each of the four lineups consisted of the facial photographs of six persons, one suspect and five fillers. In each of the two culprit-present lineups, a randomly selected face of one of the hooligans of the video the participants had seen was presented among the fillers. In each of the two culprit-absent lineups, the face of an innocent suspect was presented that the participants had not witnessed committing a crime. The innocent suspect was one of the hooligans from the video that the participants had not seen. The innocent suspect had the same hair color, hair style and stature as one of the hooligans from the video the participants had seen. For instance, if participants had seen Video 1, two randomly selected hooligans (e.g., Hooligan B and Hooligan C) from Video 1 served as the culprits in the two culprit-present lineups, while two of the hooligans from Video 2 (Hooligan A and Hooligan D in this example) served as the innocent suspects in the culprit-absent lineups. The pictures of the fillers were taken from the CVL database of Minear and Park^59^. Given that all suspects were young male adults, 20 pictures of male adults aged between 18 and 29 years were chosen as fillers. The fillers roughly resembled the culprit or innocent suspect in hair color, hairstyle and stature. Together with the fact that it was randomly determined whether participants saw Video 1 or Video 2, this procedure served to ensure that the culprits and innocent suspects differed to the same degree, on average, from the fillers in the lineup. In this way the procedure is highly ecologically valid because the situation corresponds closely to that of a real-world lineup in which the photograph of the suspect (whose status as culprit or innocent suspect is unknown to the police), stems from a different source (e.g., social media) than the photographs of the fillers (e.g., a database) and may thus differ to some degree from that of the fillers. All photographs showed the faces from a frontal view with a neutral facial expression against a black background with no clothes visible. The photographs were edited to harmonize face sizes and lighting conditions and were presented in a resolution of 142 × 214 pixels. The order of the lineups was randomized, as was the position of the suspect and the fillers in each lineup.

**Figure 3:**
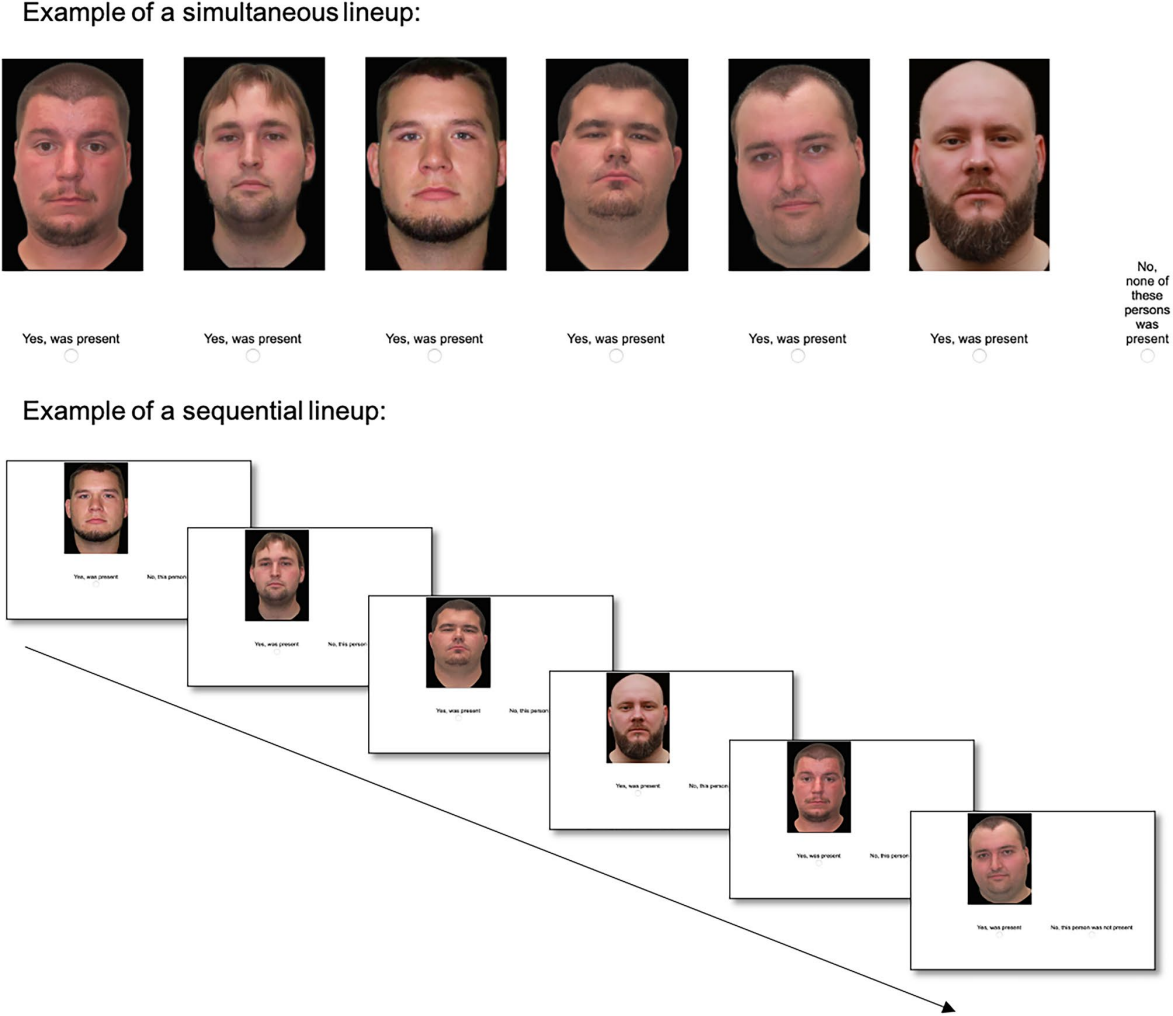
Examples of the simultaneous and sequential lineup procedures. The figure shows English translations of the German labels shown to the German participants.

It was randomly determined whether participants saw simultaneous or sequential lineups (Fig. 3). In a simultaneous lineup, the photographs of the suspect and the filler faces were presented in a row. Participants clicked on the “Yes, was present” button below a photograph if they thought that it showed one of the culprits. They clicked on the “No, none of these persons was present” button to reject the lineup. To make the procedure similar to that of a typical police lineup, participants also indicated how confident they were that their decision was correct. The participants initiated the presentation of the next lineup by clicking on a “Continue” button. In a sequential lineup, the photographs were presented successively. Participants decided for each photograph whether it showed one of the culprits or not by clicking on the “Yes, was present” button below the photograph or the “No, this person was not present” button presented at the right side of the screen, respectively. Participants had to make a decision for each of the six photographs. If a participant identified more than one person in a lineup, the last decision was used in the analysis, in accordance with standard police procedures in Germany^60^ and other jurisdictions (cf.^61–63^) and parallel to the simultaneous lineups in which participants were able to revise their decision before they clicked on the “Continue” button. To make the procedure similar to that of a typical police lineup, participants also indicated how confident they were that their decision was correct. The lineup was rejected if none of the members of the lineup was identified as one of the culprits.

### Results

For all analyses reported in this article, model fits and parameter estimates were obtained using *multiTree*^30^. One instance of the model illustrated in Fig. 1 was needed for each cell of the 2 (exposure duration: long exposure vs. short exposure) × 2 (lineup procedure: simultaneous vs. sequential) between-subjects design. The raw response frequencies are reported in Table 2.

**Table 2:** Raw response frequencies observed in Experiments 1 to 4.

	Culprit-present lineups			Culprit-absent lineups		
	Culprit identifications	Filler identifications	Lineup rejections	Innocent-suspect identifications	Filler identifications	Lineup rejections
**Experiment 1**						
Simultaneous						
Long exposure	147	94	141	38	138	206
Short exposure	64	152	166	44	157	181
Sequential						
Long exposure	140	164	58	46	212	104
Short exposure	80	193	85	57	213	88
**Experiment 2**						
Simultaneous						
Fair	156	99	129	30	140	214
Unfair	172	71	135	60	100	218
Sequential						
Fair	137	164	65	43	213	110
Unfair	177	155	52	81	182	121
**Experiment 3**						
Simultaneous						
Low culprit probability	128	78	190	28	119	249
High culprit probability	141	128	107	44	167	165
Sequential						
Low culprit probability	104	119	151	38	144	192
High culprit probability	136	164	62	56	199	107
**Experiment 4**						
Simultaneous						
Difficult to reject	142	121	135	40	163	195
Easy to reject	136	103	119	19	140	199
Sequential						
Difficult to reject	148	165	85	52	233	113
Easy to reject	125	183	82	35	191	164

**Figure 4:**
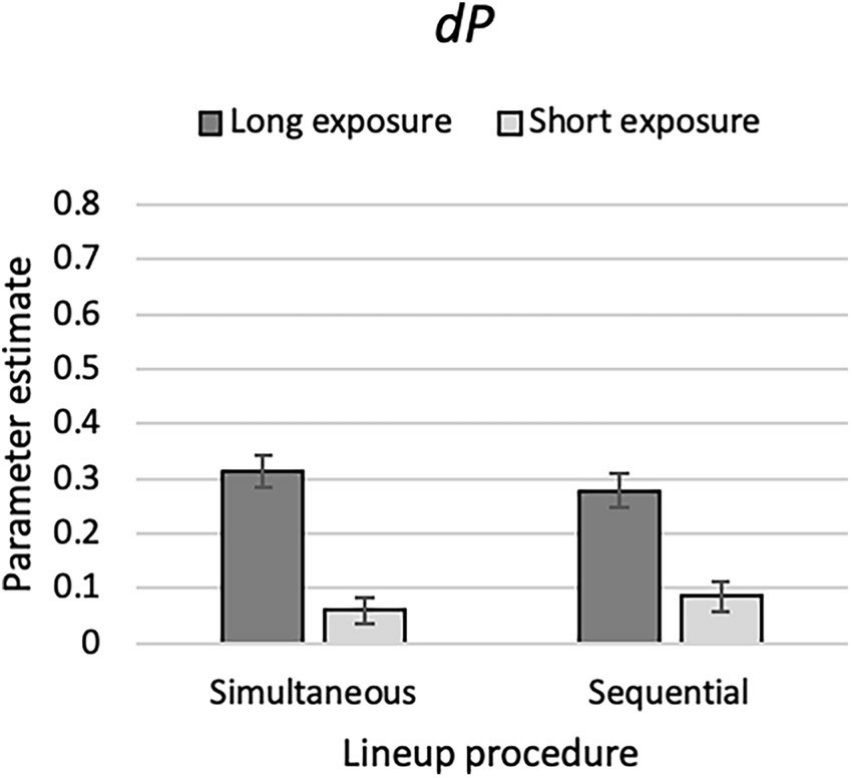
Estimates of parameter *dP* of the 2-HT eyewitness identification model representing the probability of detecting the presence of the culprit in Experiment 1 as a function of exposure duration (long exposure or short exposure) and lineup procedure (simultaneous or sequential). The error bars represent the standard errors.

Our goal was to begin with a base model that was as simple as possible. Therefore, we used whatever we could derive from the design of the studies to impose restrictions onto the 2-HT eyewitness identification model. First, it is obvious that lineup fairness must necessarily be the same across conditions because the same lineups were used in all conditions. Therefore, we set the biased-suspect-selection parameter *b* to be equal across all conditions. Second, for the same reason parameter *dA* was also set to be equal across all conditions. The base model incorporating these trivial restrictions fit the data, *G*^2^(6) = 3.91, *p* = 0.689. Multinomial processing-tree models allow to test hypotheses directly at the level of the postulated processes. For instance, the hypothesis that the culprit-presence detection parameter *dP* is higher in the long-exposure condition than in the short-exposure condition can be implemented by setting parameter *dP* to be equal between these conditions. If the model including this equality restriction provides a significantly worse fit to the data than the base model, then it is necessary to conclude that parameter *dP* differs between conditions. The estimates of the culprit-presence detection parameter *dP* are shown in Fig. 4. Parameter *dP* was significantly higher in the long-exposure condition than in the short-exposure condition, *ΔG*^2^(2) = 72.21, *p* < 0.001.

The estimates of parameters *b*, *g* and *dA* are reported in Table 3. In the simultaneous lineups, the probability of guessing-based selection (captured by parameter *g*) was significantly higher when exposure duration was short than when it was long, *ΔG*^2^(1) = 6.47, *p* = 0.011, which is to be expected given the well-known phenomenon of compensatory guessing^64–67^. Interestingly, exposure duration had no effect on guessing-based selection in the sequential lineups, *ΔG*^2^(1) = 0.03, *p* = 0.871, suggesting that the sequential presentation of the faces in the lineup prevented compensatory guessing.

**Table 3:** Parameter estimates and standard errors (in parentheses) of parameters *b*, *g* and *dA* in Experiment 1.

Lineup procedure	Exposure duration	Parameter		
		*b*	*g*	*d*A
Simultaneous	Long exposure	0.03 (0.01)	0.45 (0.02)	0.03 (0.03)
	Short exposure		0.52 (0.02)	
Sequential	Long exposure		0.75 (0.02)	
	Short exposure		0.75 (0.02)	

### Discussion

Long exposure of the culprit’s face provides ample opportunity for encoding which should increase the probability of detecting the culprit’s face in a lineup. Experiment 1 confirmed this prediction. Parameter *dP* was significantly higher in the long-exposure condition than in the short-exposure condition. The validation of parameter *dP* was thus successful; parameter *dP* sensitively reflected the manipulation of culprit-presence detection in the predicted direction.

In simultaneous lineups, parameter *g* was also affected by exposure duration. In the short-exposure condition in which culprit-presence detection was poor, guessing-based selection was more prevalent than in the long-exposure condition in which culprit-presence detection was better. This effect on parameter *g* is expected given the well-known phenomenon of compensatory guessing: people often rely on guessing to compensate for poor memory^64–67^. Note that the simplest situation in model validation is one in which only the target parameter (*dP* in the present experiment) is affected by the manipulation. This is not always possible because manipulations may have side effects. The ideal situation in such cases is one in which there is an obvious and plausible explanation of these side effects—such as compensatory guessing in the present instance. Interestingly, evidence of compensatory guessing was absent in the sequential-lineup condition. The sequential presentation of the faces may thus protect against compensatory guessing: in sequential lineups, participants may not increase their reliance on guessing when culprit-presence detection is poor because they cannot know whether the face of the culprit is yet to be presented. In simultaneous lineups, it is more obvious that the culprit cannot be detected, which may provide more favorable conditions for compensatory guessing. This hypothesis can be further explored in future research.

## Experiment 2: Biased selection of the suspect (parameter *b*)

Experiment 2 served to test the validity of parameter *b* which is assumed to represent the probability of biased selection of a culprit or an innocent suspect who stands out from the fillers. Biased suspect selection is thus assumed to occur when lineups are unfair. In Experiment 1, care was taken to create fair lineups in which the suspect did not stand out from the fillers. The fact that the estimate of parameter *b* was extremely low suggests that biased suspect selection occurred with a very low probability. In Experiment 2, this fair-lineup condition was contrasted with an unfair-lineup condition. A straightforward method of creating unfair lineups is to add a conspicuous feature such as a birthmark to the suspect’s face that distinguishes this face from the filler faces^68^. In line with this approach, unfair lineups were created by adding large birthmarks to all filler faces but not to the faces of the culprits and innocent suspects. The suspect’s face thus stood out from these fillers in that it was the only face without a birthmark. If the 2-HT eyewitness identification model is valid, then the biased-suspect-selection parameter *b* should be higher in the unfair-lineup condition than in the fair-lineup condition.

### Method

#### Sample

Participants who had not participated in Experiment 1 were recruited using the *Gapfish* research panel. We aimed at achieving about the same sample size as in Experiment 1. In total, 930 participants gave informed consent but 139 of them did not complete the study. A total of 19 datasets had to be excluded because of multiple participations. Another 16 datasets had to be excluded because participants had failed the attention check or because they had reported technical problems. The final sample included 756 participants (355 women and 401 men) with a mean age of 44 years (*SD* = 15). A sensitivity analysis with G*Power^69^ showed that with a sample size of *N* = 756, four eyewitness identification decisions, α = 0.05 and a statistical power of 1 − β = 0.95, it was possible to detect even small effects of the unfairness on the biased-selection parameter *b* of size *w* = 0.07.

#### Materials and procedure

**Figure 5:**
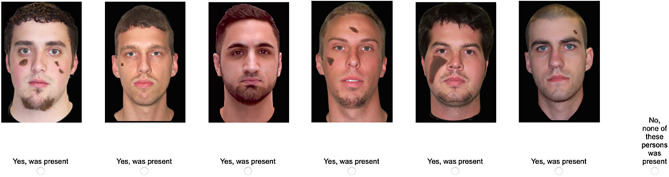
Example of an unfair simultaneous lineup used in Experiment 2. The figure shows English translations of the German labels shown to the German participants.

Experiment 2 was identical to Experiment 1, with the following exceptions. Participants saw the full-length staged-crime video used in the long-exposure condition of Experiment 1. Unfair lineups were created by digitally manipulating the filler photographs to create lineups in which all lineup members except the suspect had large birthmarks on their faces (Fig. 5). In the fair-lineup condition, participants saw the same lineups as in Experiment 1.

### Results

**Figure 6:**
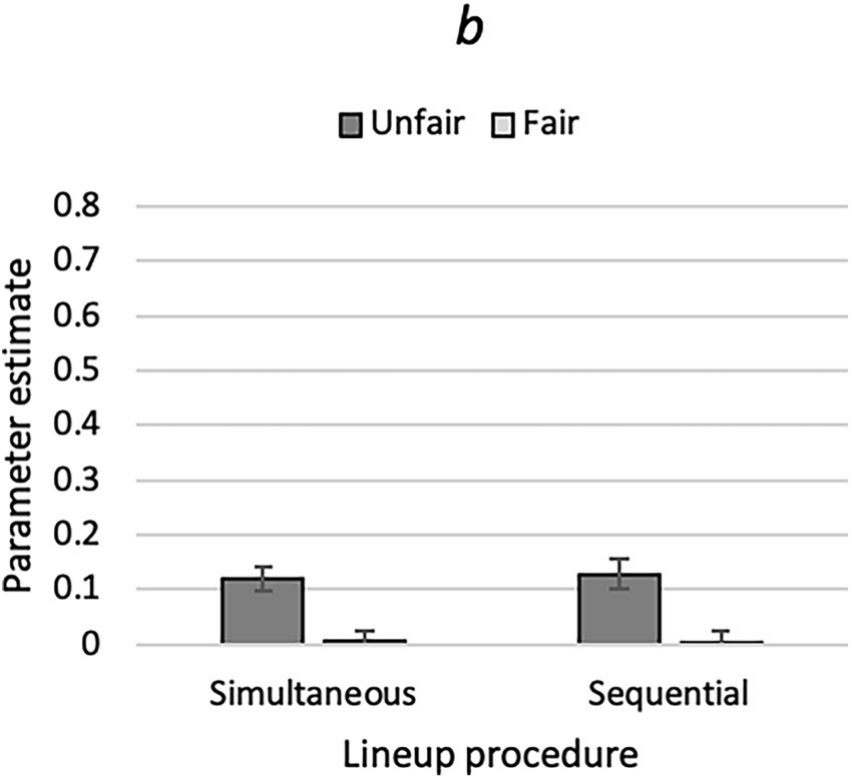
Estimates of parameter *b* of the 2-HT eyewitness identification model representing the probability of biased suspect selection in Experiment 2 as a function of the unfairness of the lineup (unfair or fair) and lineup procedure (simultaneous or sequential). The error bars represent the standard errors.

One instance of the model illustrated in Fig. 1 was needed for each cell of the 2 (lineup fairness: unfair vs. fair) × 2 (lineup procedure: simultaneous vs. sequential) design. The raw response frequencies are reported in Table 2. As in Experiment 1 and for the reason given there, parameter *dA* was set to be equal across all conditions. The base model incorporating these restrictions fit the data, *G*^2^(3) = 2.32, *p* = 0.509. The estimates of the biased-suspect-selection parameter *b* are shown in Fig. 6. Parameter *b* was significantly higher when the lineup was unfair than when it was fair, *ΔG*^2^(2) = 28.59, *p* < 0.001.

The estimates of parameters *dP*, *g* and *dA* are reported in Table 4. Culprit-presence detection (captured by parameter *dP*) did not differ as a function of lineup fairness, *ΔG*^2^(2) = 0.12, *p* = 0.940. Guessing-based selection (captured by parameter *g*) was less likely when the lineup was unfair than when it was fair, but this difference was significant only in the simultaneous lineup condition, *ΔG*^2^(1) = 7.93, *p* = 0.005, and not in the sequential lineup condition, *ΔG*^2^(1) = 0.09, *p* = 0.760.

**Table 4:** Parameter estimates and standard errors (in parentheses) of parameters *dP*, *g* and *dA* in Experiment 2.

Lineup procedure	Lineup fairness	Parameter		
		*dP*	*g*	*dA*
Simultaneous	Unfair	0.34 (0.04)	0.40 (0.02)	0.11 (0.03)
	Fair	0.35 (0.03)	0.49 (0.02)	
Sequential	Unfair	0.29 (0.04)	0.76 (0.02)	
	Fair	0.28 (0.03)	0.77 (0.02)	

### Discussion

The results support the validity of the interpretation that parameter *b* represents the process of biased suspect selection: parameter *b* was higher in the unfair conditions than in the fair conditions. In simultaneous lineups, lineup fairness also affected parameter *g*. A possible explanation is that guessing-based selection may have been reduced by the relative ease with which the fillers could be ruled out due to their large birthmarks in the unfair-lineup condition.

## Experiment 3: Guessing-based selection among the lineup members (parameter *g*)

Experiment 3 served to test the validity of parameter *g* which is assumed to reflect the probability of guessing-based selection among the lineup members. A well-established way to manipulate guessing in old-new recognition paradigms is to create the expectation (e.g., via instructions) that a stimulus is more likely to be old than new or vice versa^70^. Similarly, one-sided pre-lineup instructions insinuating that the culprit is in the lineup increase the witnesses’ willingness to guess that one of the lineup members is the culprit compared to two-sided pre-lineup instructions that emphasize the possibility that the culprit may not be in the lineup (e.g.^50,71,72^). For instance, Lindsay et al.^73^ manipulated guessing by providing either one-sided pre-lineup instructions stating that “The guilty party is in the lineup, all you have to do is pick him out” or two-sided pre-lineup instructions stating that “the guilty party may or may not be in the lineup” (p. 799). Similarly, half of the participants in Experiment 3 received the instruction that the probability of the culprit being in the lineup was high which should encourage participants to select one of the lineup members as the culprit based on guessing. The other half of the participants received the instruction that the probability of the culprit being in the lineup was low which should discourage participants from selecting one of the lineup members as the culprit based on guessing. If parameter *g* of the 2-HT eyewitness identification model is valid, then parameter *g* should be higher in the high-culprit-probability condition than in the low-culprit-probability condition.

### Method

#### Sample

Participants who had not participated in Experiments 1 and 2 were recruited using the *Gapfish* research panel. We aimed at achieving about the same sample size as in Experiments 1 and 2. In total, 896 participants gave informed consent but 108 of them did not complete the study. A total of 23 datasets had to be excluded because of multiple participations. Another 11 datasets had to be excluded because participants had failed the attention check or because they had reported technical problems. The final sample included 754 participants (336 women, 417 men, 1 diverse) with a mean age of 45 years (*SD* = 15). A sensitivity analysis with G*Power^69^ showed that with a sample size of *N* = 754, four eyewitness identification decisions, α = 0.05 and a statistical power of 1 − β = 0.95, it was possible to detect even small effects of the pre-lineup instructions on the guessing-based-selection parameter *g* of size *w* = 0.07.

#### Materials and procedure

Experiment 3 was identical to Experiment 1 with the following exceptions. Participants saw the full-length staged-crime video used in the long-exposure condition of Experiment 1. In the high-culprit-probability condition, the instructions read: *“It is ****likely**** that there is one of the culprits in each of the lineups. Therefore, you should select the ‘Yes, was present’ button that belongs to the recognized face if one of the persons feels familiar”.* Before each lineup, participants in the high-culprit-probability condition received the following reminder: *“It is likely that one of the culprits is in the lineup. You simply have to choose him”.* In the low-culprit-probability condition, the instructions read: *“It is ****unlikely**** that one of the culprits is in the lineup. Therefore, you should select the ‘Yes, was present’ button that belongs to the recognized face only if you are very certain that you have recognized the right person”.* Before each lineup, participants in the low-culprit-probability condition received the following reminder: “*It is unlikely that one of the culprits is in the lineup. Please choose someone only if you are very certain”.*

### Results

**Figure 7:**
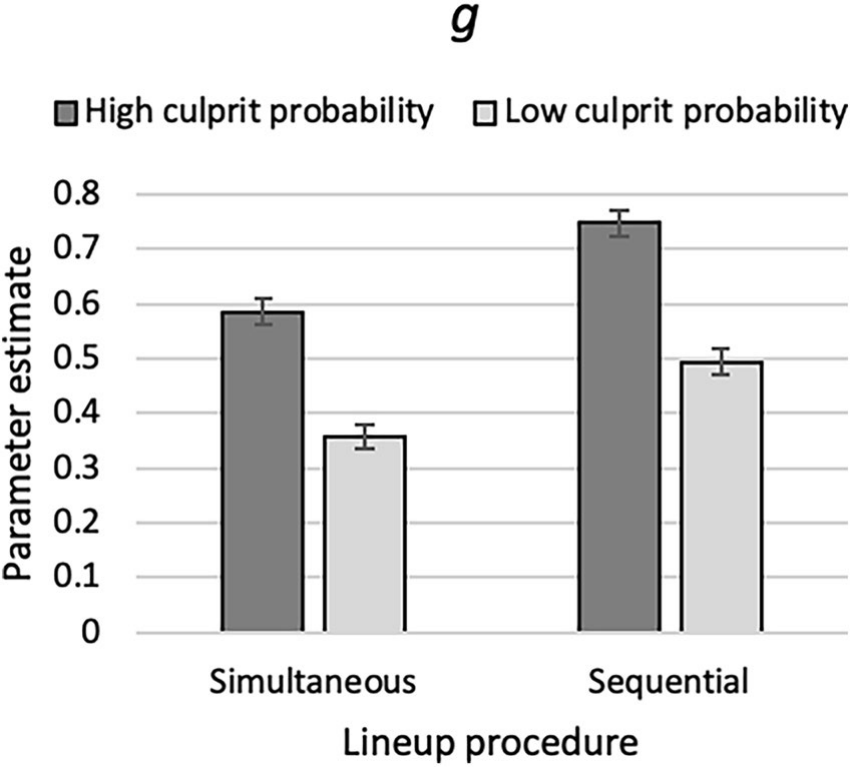
Estimates of parameter *g* of the 2-HT eyewitness identification model representing the probability of guessing-based selection among the lineup members in Experiment 3 as a function of the pre-lineup instructions (high culprit probability or low culprit probability) and lineup procedure (simultaneous or sequential). The error bars represent the standard errors.

One instance of the model illustrated in Fig. 1 was needed for each cell of the 2 (pre-lineup instructions: high culprit probability vs. low culprit probability) × 2 (lineup procedure: simultaneous vs. sequential) design. The raw response frequencies are reported in Table 2. As in Experiment 1 and for the reason given there, parameters *b* und *dA* were set to be equal across all conditions. The base model incorporating these restrictions fit the data, *G*^2^(6) = 3.85, *p* = 0.698. The estimates of the guessing-based selection parameter *g* are shown in Fig. 7. Parameter *g* was significantly higher in the high-culprit-probability condition than in the low-culprit-probability condition, *ΔG*^2^(2) = 137.36, *p* < 0.001.

The estimates of parameters *dP*, *b* and *dA* are reported in Table 5. Culprit-presence detection (captured by parameter *dP*) did not differ as a function of the pre-lineup instructions, *ΔG*^2^(2) = 4.13, *p* = 0.127.

**Table 5:** Parameter estimates and standard errors (in parentheses) of parameters *dP*, *b* and *dA* in Experiment 3.

Lineup procedure	Pre-lineup instructions	Parameter		
		*dP*	*b*	*dA*
Simultaneous	High culprit probability	0.29 (0.03)	0.03 (0.01)	0.05 (0.03)
	Low culprit probability	0.26 (0.03)		
Sequential	High culprit probability	0.27 (0.03)		
	Low culprit probability	0.19 (0.03)		

### Discussion

The results support the validity of the interpretation that parameter *g* represents the probability of guessing-based selection among the lineup members. Parameter *g* was higher when participants expected culprits in the lineups with a high rather than low probability.

## Experiment 4: Detection of the absence of the culprit (parameter *dA*)

Experiment 4 served to test the validity of parameter *dA* which is assumed to represent the probability of detecting the absence of the culprit. To manipulate parameter *dA*, the standard lineups were compared against lineups in which it could easily be detected that none of the lineup members was the culprit. In these easy-to-reject culprit-absent lineups, all lineup members, including the innocent suspect, shared a conspicuous facial feature that could be used to rule out the lineup members as culprits. These easy-to-reject lineups were compared to standard culprit-absent lineups that were difficult to reject because all lineup members, including the innocent suspect, matched the culprit in appearance. If the 2-HT eyewitness identification model is valid, the culprit-absence detection parameter *dA* should be higher when the lineup is easy to reject than when it is difficult to reject.

### Method

#### Sample

Participants who had not participated in Experiments 1 to 3 were recruited using the *Gapfish* research panel. We aimed at achieving about the same sample size as in Experiments 1 to 3. In total, 929 participants gave informed consent but 117 of them did not complete the study. A total of 22 datasets had to be excluded because of multiple participations. Another 18 datasets had to be excluded because participants had failed the attention check or because they had reported technical problems. The final sample included 772 participants (331 women, 437 men, 4 diverse) with a mean age of 46 years (*SD* = 15). A sensitivity analysis with G*Power^69^ showed that with a sample size of *N* = 772, four eyewitness identification decisions, α = 0.05 and a statistical power of 1 − β = 0.95, it was possible to detect even small effects of the ease of lineup rejection on the detection of the absence of the culprit parameter *dA* of size *w* = 0.07.

#### Materials and procedure

Experiment 4 was identical to Experiment 1 with the following exceptions. Participants saw the full-length staged-crime video used in the long-exposure condition of Experiment 1. For the easy-to-reject condition, culprit-absent lineups were created by digitally manipulating the facial photographs such that all lineup members, including the innocent suspect, had large birthmarks on their faces, just like the filler faces in the unfair-lineup condition of Experiment 2 (Fig. 5). This manipulation was designed to facilitate the detection of culprit absence. In the difficult-to-reject condition, participants saw the same culprit-absent lineups as in Experiment 1.

### Results

**Figure 8:**
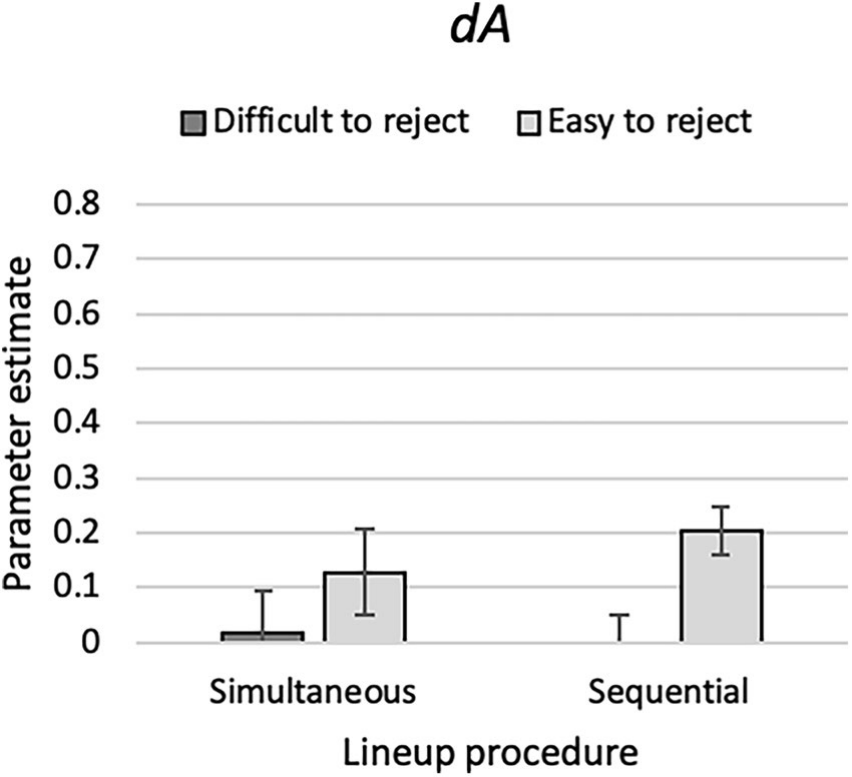
Estimates of parameter *dA* of the 2-HT eyewitness identification model representing the probability of culprit-absence detection in Experiment 4 as a function of ease of rejection of the culprit-absent lineups (difficult to reject or easy to reject) and lineup procedure (simultaneous or sequential). The error bars represent the standard errors.

One instance of the model illustrated in Fig. 1 was needed for each cell of the 2 (ease of rejection: difficult to reject vs. easy to reject) × 2 (lineup procedure: simultaneous vs. sequential) between-subjects design. The raw response frequencies are reported in Table 2. As in Experiment 1 and for the reason given there, parameter *b* was set to be equal across all conditions. The base model incorporating these restrictions fit the data, *G*^2^(3) = 5.00, *p* = 0.172. The estimates of the culprit-absence detection parameter *dA* are shown in Fig. 8. Parameter *dA* was significantly higher when the culprit-absent lineup was easy to reject than when it was difficult to reject, *ΔG*^2^(2) = 11.95, *p* = 0.003.

The estimates of parameters *dP*, *b* and *g* are reported in Table 6. The ease-of-rejection manipulation affected neither culprit-presence detection (captured by parameter *dP*), *ΔG*^2^(2) = 2.93, *p* = 0.231, nor guessing-based selection (captured by parameter *g*), *ΔG*^2^(2) = 0.38, *p* = 0.827.

**Table 6:** Parameter estimates and standard errors (in parentheses) of parameters *dP*, *b* and *g* in Experiment 4.

Lineup procedure	Ease of rejection	Parameter		
		*dP*	*b*	*g*
Simultaneous	Difficult to reject	0.30 (0.03)	0.00 (0.01)	0.52 (0.03)
	Easy to reject	0.32 (0.03)		0.51 (0.03)
Sequential	Difficult to reject	0.29 (0.03)		0.71 (0.03)
	Easy to reject	0.23 (0.03)		0.73 (0.03)

### Discussion

The results support the validity of the interpretation that parameter *dA* represents the probability of detecting the absence of the culprit. Parameter *dA* was enhanced when the culprit-absent lineups were easy to reject because all lineup members, including the innocent suspect, had the same conspicuous facial feature which could be used to rule them out as culprits.

## General discussion

Here we introduce the 2-HT eyewitness identification model that serves to measure latent processes underlying eyewitness identification performance. Before such a measurement model is applied to novel and unresolved research questions, it is important to empirically assess whether the model’s parameters sensitively reflect the processes they were designed to measure. This was done here by testing whether manipulations that can reasonably be expected to target culprit-presence detection, biased suspect selection, guessing-based selection and culprit-absence detection are sensitively reflected in the model’s parameters. In Experiment 1, parameter *dP* sensitively reflected the manipulation of exposure duration that can be assumed to affect the detection of the presence of the culprit; parameter *dP* was larger when the culprits’ faces were visible for a long time than when they were visible for only a short time. In Experiment 2, the manipulation of lineup fairness was sensitively reflected in parameter *b* that was designed to capture the biased selection of the suspect which occurs in unfair lineups; parameter *b* was larger when a conspicuous facial feature distinguished the filler faces from the suspects’ faces than when this conspicuous facial feature was absent in all faces of the lineups. In Experiment 3, the manipulation of the pre-lineup instructions was sensitively reflected in parameter *g* that was designed to measure guessing-based selection; parameter *g* was larger when the participants were led to expect culprits in the lineups with a high rather than low probability. The effects demonstrated in Experiments 1 to 3 are closely aligned with well-established findings in the literature^40,44,46,48,50,53,68,70,72–81^. This reflects the deliberate strategy to use, for the purpose of validation experiments, manipulations that are well understood so that one can be certain as to which processes and, hence, which model parameters they should affect. This strategy was not readily available for the process of the detection of culprit absence, reflected in parameter *dA*, possibly because other approaches for analyzing lineup data do not specifically postulate a separate process for culprit-absence detection so that this process is less well studied. Therefore, an intentionally trivial manipulation was used in Experiment 4: culprit-absence detection should be easier when members of culprit-absent lineups can be easily rejected based on a conspicuous facial feature that distinguishes them from the culprit. This ease-of-rejection manipulation was reflected in parameter *dA* that was designed to capture the detection of the absence of the culprit; parameter *dA* was higher when the members of the culprit-absent lineups were easy to reject than when they were difficult to reject.

It is a distinguishing feature of the 2-HT eyewitness identification model that it differentiates between two types of detection processes: the detection of culprit presence and the detection of culprit absence. Typical analyses of lineup data provide a single accuracy measure that is intended to reflect the witnesses’ ability to distinguish between culprit presence and absence. The 2-HT eyewitness identification model goes one step further by allowing to measure these two processes separately. The results of the present series of validation experiments suggest that these two types of detection processes can be manipulated independently of each other. The manipulation of exposure duration affected the culprit-presence detection parameter *dP* but not the culprit-absence detection parameter *dA,* whereas the manipulation of the ease with which culprit-absent lineups can be rejected influenced *dA* but not *dP*. Including a separate parameter for culprit-absence detection in a measurement model may help to stimulate thinking about how lineup procedures may help to improve the process of culprit-absence detection.

Another distinguishing feature of the present model is that it differentiates between two forms of non-detection-based processes. Parameter *g* reflects the process of selecting a lineup member based on guessing, while parameter *b* reflects the biased selection of a suspect who stands out from the fillers in an unfair lineup. This distinction is, of course, not entirely new, but the qualitative distinction between the two types of non-detection-based selection processes may be emphasized if they are distinguished in a measurement model. In the lineups used in Experiments 1, 3 and 4 and in the fair-lineup condition of Experiment 2, care was taken to create fair lineups by selecting fillers that resembled the culprits and the innocent suspects in hair color, hairstyle and stature and by making sure that the photographs of the fillers could not be distinguished from those of the culprit and the innocent suspect based on head size or lighting conditions. When lineups are fair, the biased-suspect-selection parameter *b* can be expected to be close to zero, which was confirmed by the present experiments. Experiment 2 shows that the 2-HT eyewitness identification model is also able to detect biased suspect selection in unfair lineups. We consider it a strength of this model that it allows measuring biased suspect selection in unfair lineups directly based on the eyewitness identification data of interest as opposed to having to rely on indirect data from a separate mock-witness task for determining lineup fairness^82^.

**Figure 9:**
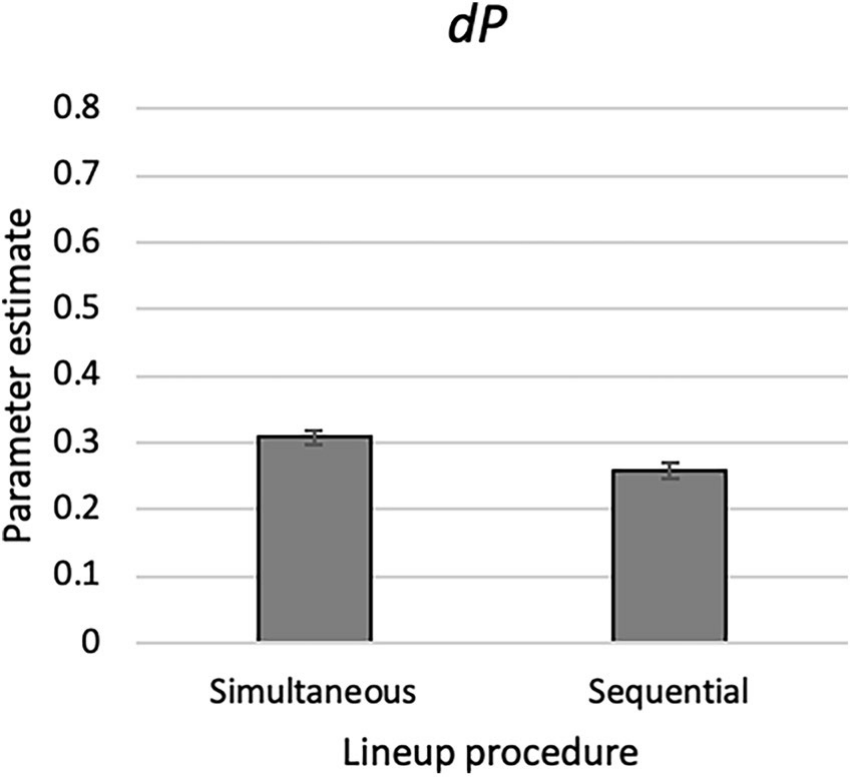
Estimates of parameter *dP* of the 2-HT eyewitness identification model representing the probability of detecting the presence of the culprit in simultaneous and sequential lineups, respectively. The estimates are based on the data of all four experiments, excluding the short-duration condition of Experiment 1. The difference in *dP* between the simultaneous conditions and the sequential conditions was small (Δ*dP* = 0.05). The error bars represent the standard errors.

The main purpose of including both simultaneous and sequential lineups in the present experiments was to test whether the 2-HT eyewitness identification model validly reflects eyewitness identification processes in both types of lineups. Indeed, manipulations of exposure duration, lineup fairness, pre-lineup instructions and ease of rejection were sensitively reflected in culprit-presence detection, biased suspect selection, guessing-based selection and culprit-absence detection for simultaneous and sequential lineups, which supports the model’s validity for measuring the latent processes underlying eyewitness identification decisions in simultaneous and sequential lineups. Beyond this main purpose it seems interesting to explore which type of lineup procedure is superior in terms of culprit-presence detection. A superiority of sequential lineups has been postulated based on the diagnosticity ratio^44^ which, however, has been criticized for confounding the ability to distinguish between a culprit and an innocent suspect and response bias^20^. SDT-based analyses have sometimes shown equivalent performance of simultaneous and sequential lineups^13,37,39,47,83,84^ and sometimes a superiority of simultaneous over sequential lineups^11,38,62,85–88^. In all experiments reported here, the estimates of the culprit-presence detection parameter *dP* were slightly but consistently higher in the simultaneous-lineup conditions than in the sequential-lineup conditions. This difference in parameter *dP* was not statistically significant in any of the individual experiments, but when we combined the data from all experiments (Fig. 9), *dP* was significantly larger in simultaneous than in sequential lineups (*ΔG*^2^[1] = 10.13, *p* = 0.001; details of this analysis are reported in the Supplementary Materials that can be found in the OSF repository). This result nicely converges with those of SDT-based analyses: there is an advantage of simultaneous compared to sequential lineups in terms of culprit-presence detection, but the advantage is small and can be reliably observed only when the sample sizes are large enough to guarantee sufficient statistical power to detect such small effects.

This convergence is not too surprising because the present 2-HT eyewitness identification model was designed to separate, among other things, culprit-presence detection (parameter *dP*) from guessing-based selection (parameter *g*), which is conceptually similar to the distinction between sensitivity (*d*') and response bias (*c*) in SDT^89^. A more surprising finding is that, in the experimental paradigm used here, the estimate of the guessing-based selection parameter *g* was consistently higher in the sequential than in the simultaneous lineups. At first glance, this may seem unexpected because it has often been found that sequential lineups lead to more conservative responding than simultaneous lineups (e.g.^83^). In the present experiments, the higher tendency towards guessing that the culprit is in the lineup is already apparent at the level of the raw response probabilities: the mean rate of lineup identifications was consistently higher in the sequential lineups than in the simultaneous lineups (0.77 to 0.55 in Experiment 1, 0.65 to 0.54 in Experiment 2, 0.77 to 0.54 in Experiment 3 and 0.72 to 0.57 in Experiment 4). The most plausible explanation of the discrepancy of the data reported here to the data obtained in previous studies is that we explicitly followed standard police protocols by continuing the presentation of the sequential lineup even after an early identification^60–63^; only the last identification was used. By contrast, in many previous studies the *only-the-first-yes-counts* rule was used. Horry et al.^62^ have demonstrated that only-the-first-yes-counts instructions systematically reduce the rate of positive identifications in sequential lineups by discouraging participants from guessing. This is plausible at a psychological level: participants may well shy away from using their only identification option when they cannot know, at the time of their decision, whether better options will follow. These findings suggest that the standard police protocol applied here may induce a particularly pronounced tendency to select a lineup member based on guessing in sequential lineups: even if a lineup member was selected at some point, a later lineup member could still be selected if the witness came to the conclusion that the later lineup member provided a better match to their memory for the culprits.

A limitation which the present approach has in common with other approaches is that it allowed us to estimate the different processes underlying eyewitness identification decisions only at the group level. Due to the small number of responses, it would be difficult to estimate parameters at the individual level^90^. As the participants saw four lineups each, only four data points per participant were collected. Compared to other paradigms designed to collect data for MPT models (e.g.,^21,22,25,27,91^), this is a comparatively small number of data points per participant. However, using a larger number of lineups would go against ecological validity because crimes with multiple culprits typically involve only a small number of culprits^92^.

Another limitation is that the 2-HT eyewitness identification model in the present version accounts only for lineup identifications and rejections but not for confidence judgements. Accounting for confidence judgements would thus require an extension of the model (cf.^35,93–98^). A more general point is that MPT models are based on a threshold concept, assuming that observed behaviors are the result of discrete mental states rather than continuously distributed variables such as an assumed memory strength variable which is posited to underly recognition decisions in SDT-based approaches^29^. With respect to such recognition decisions, some researchers have argued that the threshold assumption of MPT models is inconsistent with the available empirical evidence^99,100^, but others have shown that both SDT-based and threshold models can account for recognition memory performance^34,35,89,93–98,101^. On the whole, it is important to note that all models are necessarily simplifications of the complexities of reality. The assumption of different mental states and of thresholds that have to be crossed to get from one state to another (inherent in all threshold models and, thus, in the 2-HT eyewitness identification model) is a simplification, but so is, for instance, the assumption of equal-variance, normal distribution of an assumed signal strength variable in models based on signal detection theory^89^. The question is not which set of simplifications is correct because all simplifications of the complexities of reality must necessarily be false. Instead, the question is which set of simplifications is sufficiently useful. Here we introduced a novel MPT model to the field of eyewitness identification research which we hope may turn out to be useful in that it makes it possible to measure the four latent cognitive processes of culprit-presence and culprit-absence detection, biased selection and guessing-based selection using the *whole* 2 × 3 data matrix of the typical lineup identification task (Table 1). When introducing a novel MPT model, it is an important first step to experimentally validate the interpretation of the model parameters by providing an empirical test of whether the model parameters capture the processes they were designed to measure, which is the purpose of the present series of experiments. The theoretical and practical usefulness of threshold models in the field of eyewitness identification decisions can only be determined in the long run and thus has to be evaluated based on future research.

## Conclusions

The purpose of the present study was to introduce, and to provide an experimental validation of, the novel 2-HT eyewitness identification model that takes into account the full 2 × 3 structure of lineup data. The present results support the validity of the model for analyzing lineup data. Now that the model parameters have been demonstrated to sensitively reflect the effects of well-established and obvious manipulations of the latent processes underlying eyewitness identification decisions in simultaneous and sequential lineups, the model can be used in future studies to measure the latent processes underlying the witnesses’ decisions in lineups, for example, to test novel hypotheses.

## Supplementary Information

Supplementary Information.

## Data Availability

The datasets generated and analyzed during the current study, as well as supplementary materials are available in the OSF repository, https://osf.io/qbzs2/.
