# Supplementary material for: Experimental validation of a multinomial processing tree model for analyzing eyewitness identification decisions
Source: Sci Rep. 2022 Sep 16;12:15571. doi: 10.1038/s41598-022-19513-w (PMC9481595; doi:10.1038/s41598-022-19513-w)
Supplement: Supplementary file 1 — Supplementary Information. [file 41598_2022_19513_MOESM1_ESM.docx]

# Supplementary information on the analysis of comparing culprit-presence detection between simultaneous and sequential lineups across all experiments in “Experimental validation of a multinomial processing tree model for analyzing eyewitness identification decisions” by Winter, Menne, Bell, & Buchner

In all experiments reported in the manuscript, the estimates of parameter *dP,* representing the probability of detecting the presence of the culprit, were consistently higher in the simultaneous-lineup conditions than in the sequential-lineup conditions. However, when the hypothesis of a simultaneous superiority effect was formally tested by equating parameter *dP* across simultaneous and sequential-lineup conditions, the analyses showed that culprit-presence detection did not differ significantly between simultaneous and sequential lineups in Experiment 1, *ΔG^2^*(2) = 1.33, *p* = .513, Experiment 2, *ΔG^2^*(2) = 3.30, *p* = .192, Experiment 3, *ΔG^2^*(2) = 3.76, *p* = .153, and Experiment 4, *ΔG^2^*(2) = 5.97, *p* = .051. To further investigate this descriptive difference, we combined the data of all four experiments in a cross-experimental analysis. As a starting point, we used a base model that combined all assumptions of the individual base models used in Experiments 1 to 4. This model fit the data, *G^2^*(18) = 15.07, *p* = .657. We then made use of the fact that the participants of Experiments 2 to 4 had seen the full video of the crime, that is, the video of the long-exposure condition of Experiment 1. Therefore, we assumed that for participants who had seen the full video and were tested with the same (simultaneous or sequential) lineup procedure, parameter *dP* should not differ across experiments. This assumption was compatible with the data, *ΔG^2^*(12) = 15.68, *p* = .206. The new base model incorporating this assumption had one parameter for the detection of the culprit in simultaneous lineups and one parameter for the detection of the culprit in sequential lineups across all four experiments (excluding the short-duration condition of Experiment 1) and fit the data, *G^2^*(30) = 30.75, *p* = .428. The restriction that parameter *dP* is identical for simultaneous and sequential lineups resulted in a significant misfit, *ΔG^2^*(1) = 10.13, *p* = .001. This leads to the conclusion that culprit-presence detection is significantly more likely in simultaneous than in sequential lineups.
